# Genome-Wide Screening for Pathogenic Proteins and microRNAs Associated with Parasite–Host Interactions in *Trypanosoma brucei*

**DOI:** 10.3390/insects13110968

**Published:** 2022-10-22

**Authors:** Zhiyuan Yang, Mai Shi, Xiaoli Zhang, Danyu Yao

**Affiliations:** 1 School of Artificial Intelligence, Hangzhou Dianzi University, Hangzhou 310018, China; 2 School of Biomedical Sciences, The Chinese University of Hong Kong, Hong Kong 999077, China; 3 School of Physics and Key Laboratory of Molecular Biophysics of the Ministry of Education, Huazhong University of Science and Technology, Wuhan 430074, China; 4 School of Biological Science and Medical Engineering, Beihang University, Beijing 100083, China

**Keywords:** *Trypanosoma brucei*, microRNA, bioinformatics, parasite–host interaction

## Abstract

**Simple Summary:**

Tsetse flies are blood-sucking insect vectors belonging to the order Diptera and are widely distributed in the areas of sub-Saharan Africa. These vectors can transmit the pathogenic parasite *Trypanosoma brucei* (*T. brucei*) which can cause a disease called African trypanosomiasis. Currently, discovering effective therapeutic drugs and vaccines with minor side effects is still one of the ongoing efforts to treat the disease as well as to prevent the epidemic. Genome technology has recently played an important role in uncovering the molecular mechanisms of many vector-borne diseases, which facilitates the identification of potential drug targets. To better understand the pathological contribution of parasite–host interactions to the disease, we studied the genomic and transcriptomic profiles of *T. brucei*. We identified a panel of pathogenic proteins and microRNAs supported by their molecular functions in *T. brucei* for the first time. Our study may pave a new avenue for designing preventive and therapeutic strategies to control this insect vector.

**Abstract:**

Tsetse flies are a type of blood-sucking insect living in diverse locations in sub-Saharan Africa. These insects can transmit the unicellular parasite *Trypanosoma brucei* (*T. brucei*) which causes African trypanosomiasis in mammals. There remain huge unmet needs for prevention, early detection, and effective treatments for this disease. Currently, few studies have investigated the molecular mechanisms of parasite–host interactions underlying African trypanosomiasis, mainly due to a lack of understanding of the *T. brucei* genome. In this study, we dissected the genomic and transcriptomic profiles of *T. brucei* by annotating the genome and analyzing the gene expression. We found about 5% of *T. brucei* proteins in the human proteome, while more than 80% of *T. brucei* protein in other trypanosomes. Sequence alignment analysis showed that 142 protein homologs were shared among *T. brucei* and mammalian genomes. We identified several novel proteins with pathogenic potential supported by their molecular functions in *T. brucei*, including 24 RNA-binding proteins and six variant surface glycoproteins. In addition, 26 novel microRNAs were characterized, among which five miRNAs were not found in the mammalian genomes. Topology analysis of the miRNA-gene network revealed three genes (RPS27A, UBA52 and GAPDH) involved in the regulation of critical pathways related to the development of African trypanosomiasis. In conclusion, our work opens a new door to understanding the parasite–host interaction mechanisms by resolving the genome and transcriptome of *T. brucei*.

## 1. Introduction

Tsetse flies are a type of blood-sucking insect that live in diverse locations in sub-Saharan Africa. Unlike many other insect vectors, tsetse flies feed solely on and proliferate by vertebrate blood. Tsetse flies are well-known disease vectors as they carry and transmit pathogenic protozoan *Trypanosoma brucei* (*T. brucei*) [1]. *T. brucei* is a parasite that causes African trypanosomiasis and this disease can be divided into Human African trypanosomiasis (HAT, also known as “sleeping sickness”) and Animal African trypanosomiasis (AAT) [2]. This parasite is a unicellular eukaryote spread through the bite of tsetse flies. *T. brucei* undergoes a series of complex differentiation and reproduction steps to inhabit the blood of mammalian hosts [3], where they complete major morphological and physiological changes during proliferation. This parasite has developed special cellular and molecular characteristics, which make it a model organism of interest to scientists [4]. Although *T. brucei* infection has been widely studied, there is no vaccine available to prevent this tropical disease [5]. Therefore, the analysis of *T. brucei* genome remains an important approach for the prevention of African trypanosomiasis in the foreseeable future.

More than ten years have passed since the first release of *T. brucei* genome annotation in 2010 [6]. In 2015, Parsons et al. applied profiling data to identify 225 coding sequences and found most of them were not yet annotated as protein-coding genes [7]. In 2022, Glockzin et al. analyzed the *T. brucei* genome and identified adenine transferase APRT, which is involved in adenosine salvage pathway [8]. Nevertheless, the functions of 5709 proteins in *T. brucei* remain inconclusive, which calls for a systematical re-annotation of *T. brucei* proteome. With the progress of genome technology, many bioinformatic tools have been developed [9] and successfully applied to annotate proteins with unknown functions in species such as *Lutzomyia longipalpis* [10] and *Chlamydomonas reinhardtii* [11]. The increased knowledge and advanced tools provide an unprecedented opportunity for the re-annotation of *T. brucei* proteins.

Few studies have investigated the role of microRNA (miRNA) in the molecular mechanisms of trypanosome infection. MiRNA is a class of small non-coding RNA molecules with 18–30nt length. The major function of miRNA is regulating protein expression in the post-transcriptional process. Recent studies have suggested the regulator roles of miRNAs during pathogen–host interactions [12]. Benefitting from the development of specific tools for in silico miRNA prediction, scientists have identified novel miRNAs from expressed sequence tags (ESTs) in *Glossina morsitans* [13] and *Cryptosporidium parvum* [14], based on conserved characteristics of miRNA across different species.

In this work, we studied the genomic and transcriptomic profiles of *T. brucei* to identify protein-coding genes and miRNAs. Protein functions were annotated and pathogenicity-related proteins were investigated. MiRNAs specific in *T. brucei* were also identified and their potential pathogenic mechanisms were discussed. By uncovering the function of proteins and microRNAs in *T. brucei*, we believe our work may provide a global picture for a better understanding of the genome and transcriptome of the parasite as well as its interaction with the hosts.

## 2. Materials and Methods

### 2.1. Genome and Database

**Figure 1:**
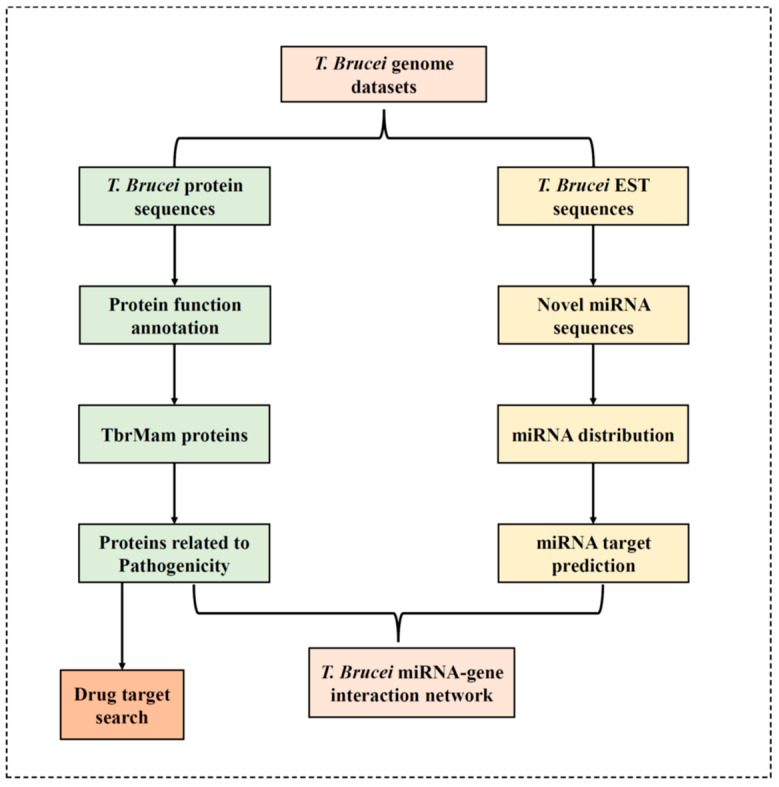
Workflow in this study. Our work was divided into two main parts: protein-coding gene analysis and miRNA analysis.

The most intensively studied trypanosome subspecies is *Trypanosoma brucei brucei* (abbreviated as *T. brucei* in this manuscript). A total of 5576 EST sequences of *T. brucei* were retrieved (20 June 2022) from NCBI database (https://www.ncbi.nlm.nih.gov/nucleotide/). The genome annotation (Assembly ID:ASM244v1) of involved species was downloaded (20 June 2022) from UniProt database (https://www.uniprot.org) [15]. All known miRNAs were collected from miRBase [16]. The workflow of this study was shown in Figure 1.

### 2.2. Protein Function Annotation

A proportion of 65.2% (5709/8758) proteins in the current version (Assembly ID:ASM244v1) of the *T. brucei* genome were annotated with “uncharacterized protein” or “hypothetical protein” (UCP). We applied a two-step strategy to re-annotate these proteins. We first performed homology searching by sequence alignment, a traditional way to predict protein function [11]. Pairwise sequence alignment was conducted (20 June 2022) between *T. brucei* and other animals using BLAST (version 2.13.0) with a cutoff E-value = 1 × 10^−6^. Because some sequence alignment similarities were relatively low, their homology was ambiguous and needed to be checked by secondary structure similarity. Next, proteins with homologs not identified by BLAST were then subject to SSEalign [17], a tool of homology detection based on the secondary structure of proteins. The statistics of resulted protein homologs were calculated and plotted.

### 2.3. Identification of TbrMam Proteins

We searched for protein homology between *T. brucei* and mammals, given mammals are the main hosts of trypanosome. Based on proteome annotation details in UniProt database, we selected ten well-studied species (*homo sapiens*, *Mus musculus*, *Rattus norvegicus*, *Bos taurus*, *Canis familiaris*, *Equus caballus*, *Cavia porcellus*, *Oryctolagus cuniculus*, *Sus scrofa*, *Rhesus macaque*) as representative mammals, to compare their protein profiles with *T. brucei* proteins using BLAST, respectively. Proteins with BLAST E-values less than 1 × 10^−6^ were considered to be present in both *T. brucei* and mammals. We denoted these shared proteins as TbrMam proteins in this study. Metascape (www.metascape.org) is a web tool that provides a comprehensive gene annotation in the system level for biologists [18]. Functional enrichment analysis of these TbrMam proteins was then performed (10 July 2022) by Metascape to find the enriched molecular pathways and ontology. ShinyGO is an application developed based on R programming and integrated with many genomic and biological pathway resources [19]. Genes that encode the TbrMam proteins were also analyzed by ShinyGO to characterize their genomic specificity.

### 2.4. Proteins Related to Pathogenicity

Trypanosome has evolved various mechanisms for survival in hosts. Three classes of proteins were reported for trypanosome pathogenicity [20] and were scrutinized in this study:

(1) RNA-binding protein (RBP): Trypanosome is deficient in specific promoters of protein-coding genes and is highly reliant on RBPs to control its RNA fate [21]. RBPs are critical to post-transcriptional gene regulation. Therefore, characterization of RBP is a key premise for elucidating their pathogenic functions;
(2) Variant surface glycoprotein (VSG): VSGs are major surface antigens recognized by the host immune system during trypanosome infection [22]. The modulation of trypanosomes can prevent long-lasting immunity in the host;
(3) Phosphatidylinositol 3 Kinase (PI3K): PI3K is a heterodimeric complex composed of a regulatory subunit and a catalytic subunit. PI3K is a lipid kinase that regulates cellular processes such as proliferation, differentiation and survival in the trypanosome. A previous report suggested that this protein is essential for autophagosome formation of trypanosome [23].

### 2.5. miRNA Identification

The miRNAs play crucial roles in various diseases. Some miRNAs were selected to target those “undruggable proteins” and approved for pre-clinical trials [24]. Although many practical difficulties need to be overcome, the miRNA-mediated method showed a possible future treatment for some diseases [25]. This type of RNA is highly conserved among different animals; thus, we could identify novel miRNAs in new animals by homolog searching. The EST-based method has been reported as a standard practice to identify novel miRNAs in the studied species [13,14]. Bowtie is an excellent tool for the alignment of short sequences and RNAfold is an effective tool for predicting RNA structure; thus, these tools and corresponding parameters were also applied in this project. We aligned all known animal miRNAs against EST sequences of *T. brucei* by tool Bowtie [26] with the toleration mismatch ≤ 2. The frequency of minimum free energy (FMFE) of matched sequence fragments was then predicted by RNAfold [27]. A cutoff FMFE ≤ 0.05 was used to determine the possibility of predicted structures. The remaining sequence fragments were regarded as novel miRNAs in this study. Furthermore, the identified miRNAs were then compared with their homologs in other species, and the homolog distribution was plotted.

### 2.6. Homolog Distribution of Identified miRNAs

Homolog distribution could help the researcher better observe the function of studied molecules. The identified miRNAs were compared with their homologs in other species and the homolog distribution was drawn. Because the mammal is the main host of *T. brucei*, all known mammalian miRNAs were compared with identified miRNAs in this study. The *T. brucei* unique miRNAs are then picked out for further analysis.

### 2.7. Network Analysis

The miRWalk [28] and miRTarBase [29] are commonly-used RNA databases including a large number of miRNA function information, the targeted genes of the identified miRNAs were then predicted by these two databases. The Cytoscape is a good tool for drawing the molecule–molecule interaction network. The predicted target genes, as well as the miRNAs, were then used to construct a miRNA-gene network visualized by Cytoscape [30]. To identify reliable biomarkers in the miRNA-gene network, we analyzed the nodes of the network using the Cytoscape plug-in “NetworkAnalyzer”, which is a common toolkit for analyzing the network topology. We selected several critical miRNAs and explored their molecular functions.

### 2.8. Potential Drug Search

In 2021, around 750 human cases of trypanosome-induced diseases were reported by World Health Organization [31]. Currently, available drugs for African trypanosomiasis are relatively unsatisfying. Some drugs have been reported to have high levels of toxicity that resulted in fatal side effects; thus, searching for potential new drugs is an urgent need. We searched (28 August 2022) for potential drugs for African trypanosomiasis by Drugbank database (http://www.drugbank.ca/). The drug names and their targets were also recorded.

## 3. Results

### 3.1. Homolog Distribution of Proteins

**Figure 2:**
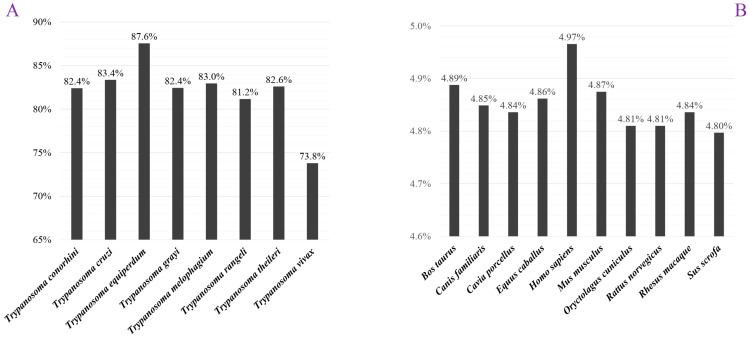
Homolog distribution of *T. brucei* proteins. (**A**) The homolog percentage of *T. brucei* when compared with other trypanosomes; (**B**) the homolog percentage of *T. brucei* when compared with other mammals.

We compared the proteins of *T. brucei* with those in other animals by sequence alignment. On one hand, we found 87.6% of the *T. brucei* proteins had homologs in *Trypanosoma equiperdum*, while 73.8% of *T. brucei* proteins shared homology with *Trypanosoma vivax* (Figure 2A). On the other hand, compared with previous results in other parasites, such as Leishmania [32], the protein homolog percentage of the trypanosome was relatively higher. The higher proportion of protein homologs between trypanosomes indicated they have evolved more similar physiological characteristics in survival.

Because mammal is the main host of *T. brucei*, we also compared the protein homology between *T. brucei* and each mammal, respectively. A previous study showed that most vertebrates and invertebrates shared at least 30% protein homologs analyzed by sequence alignment [33]. However, less than 5% of proteins of *T. brucei* could be found homologs in studied mammals (Figure 2B). For example, only 4.97% *T. brucei* proteins had homologs found in *homo sapiens* (human), while only 4.80% of proteins could be found in *Sus scrofa* (boar). We further compared the similarity of protein homologs between *T. brucei* and *homo sapiens* and found the protein similarities between the two species were mostly enriched in 35~40% (Figure S1). Most of the protein similarity was below 60%, while a few similarities were higher than 80%. The trypanosome was significantly different from the mammalian host in the proteome aspect, indicating that most proteins in the trypanosome could be used as drug target candidates for the treatment.

### 3.2. Functional Enrichment of TbrMam Proteins

Mammals are the main hosts of this trypanosome; thus, the protein homologs among *T. brucei* and ten common mammals were compared by BLAST. We detected a set of 142 proteins present in both *T. brucei* and all studied mammalian genomes. These 142 proteins formed the TbrMam proteins. Enrichment analysis revealed that the term “Neutrophil degranulation” (R-HSA-6798695) was significantly over-represented in TbrMam proteins with a *p*-value = 1.48 × 10^−11^ in the category of “Reactome Gene Sets” (Table 1). It was reported that neutrophils could enhance early infection of *T. brucei* [34]; thus, these proteins may function as pathogenic factors that may attack the host immune system. Besides, the term “intracellular protein transport” (GO:0006886) was significantly enriched in TbrMam proteins with a *p*-value = 5.62 × 10^−11^ in the category of “GO Biological Process”, which suggested that *T. brucei* may use these proteins to acquire essential nutrients from mammalian hosts.

**Figure 3:**
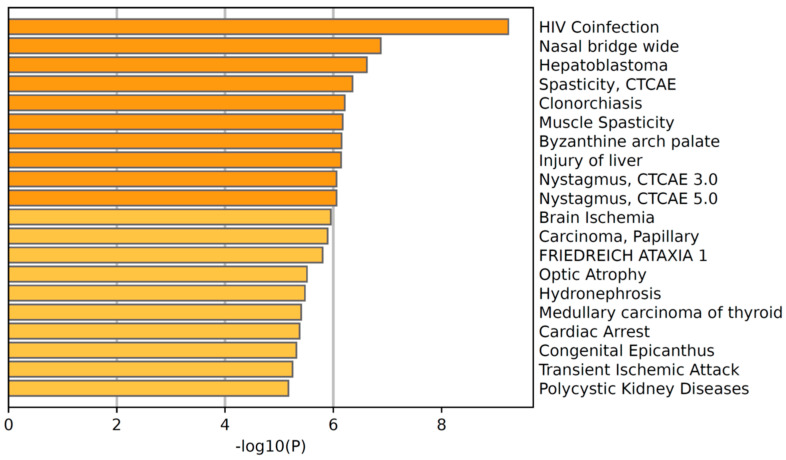
Associated disease enrichment of TbrMam proteins by Metascape tools. This figure only shows the top 20 terms.

We further investigated the disease category associated with the TbrMam proteins by Metascape tool to identify which disease was related to African trypanosomiasis. We listed the top 20 enriched diseases in Figure 3, where the term “HIV coinfection” was the most significant associated disease (*p*-value = 6.3 × 10^−10^). Interestingly, trypanosome infection is always accompanied by HIV coinfection and the phenomenon is regarded as a clinical event of great relevance [35,36]. Scientists have reported that the presence of an intracellular pathogen (such as trypanosome) could impair HIV-1 transduction in the cellular experiment in vitro [37]. On the other hand, HIV aspartyl peptidase inhibitors could also act on pathogenic trypanosome [38]. We believe that other enriched diseases could also be associated with trypanosome infection, which warrants a future investigation.

### 3.3. Genomic Specificity of TbrMam Genes

**Figure 4:**
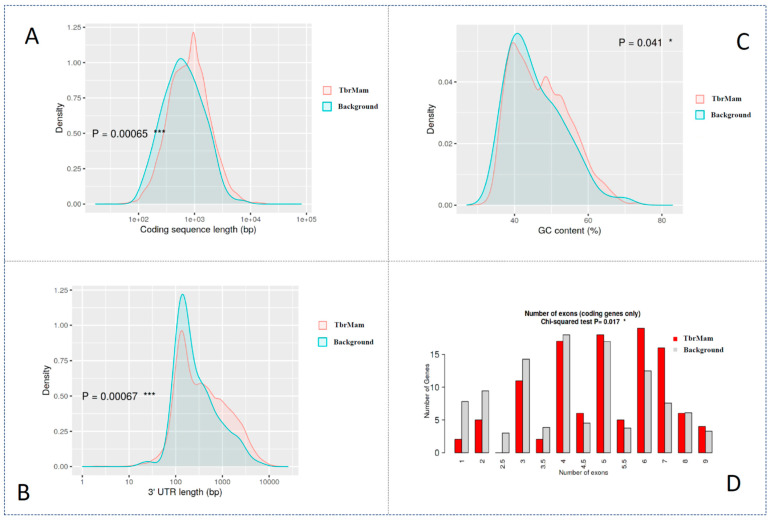
Genomic specificity of identified TbrMam genes when compared with the whole genome. (**A**) Comparison of coding sequencing length; (**B**) comparison of 3′-UTR length; (**C**) comparison of GC content; (**D**) comparison of exon numbers, the red bars indicate the TbrMam protein, while the gray bars indicate the background proteins. The asterisk symbol indicated the significance of statistical genomic specificity in the figures.

We applied ShinyGO to analyze the genomic specificity of *T. brucei* genes that encode the TbrMam proteins. We compared four aspects: exon number, coding sequence length, GC content and 3′-UTR length (Figure 4). When statistically comparing exon numbers between TbrMam genes and the rest genes in the genome, a significant *p*-value (0.07) was obtained by the Chi-squared test. Similarly, a significantly low *p*-value (0.00065) was calculated when statistically comparing the coding sequence length by Student’s *t*-test. These results indicated that our identified TbrMam genes could share strong transcription characteristics with other genes, which might be involved in the survival of *T. brucei* in the host. Moreover, an extremely low *p*-value (0.00067) was observed when statistically comparing 3′-UTR length, while an acceptable *p*-value (0.041) was obtained when statistically comparing GC content. Because 3′-UTR is the binding region of miRNA, the associated 3′-UTRs of these genes might regulate the gene expression in the trypanosome–host interaction.

### 3.4. Proteins Related to Pathogenicity

Trypanosome has evolved many mechanisms for survival in the host. Three types of proteins were reported for *T. brucei* pathogenicity (Table 2). The details of these proteins are discussed as follows.

Variant surface glycoprotein (VSG) is an important protein that has long been thought to be activated by the hydrolysis of its glycolipid membrane anchor [39]. In this study, we identified six novel VSGs in *T. brucei* by homology searching. All these VSGs showed an e-value = 0 in the sequence alignment, which is the minimum value in BLAST tool. Five VSGs shared an identity > 99%, suggesting that they had almost the same amino acid sequences. These results indicated that our identified VSGs were very reliable in *T. brucei*.

RNA-binding proteins (RBPs) play a particularly important role in regulating gene expression in trypanosomes [40]. Therefore, characterization of the RNA molecules bound by RBPs represents a key step in elucidating their function. Using sensitive database searching, a set of 24 novel RBPs were identified in *T. brucei*. Most of these RBPs are highly similar to known RBPs with an alignment identity >90%. Especially, we found that 16 RBPs showed an alignment identity = 100%, meaning that the identified RBPs are exactly the same as known sequences. These results also indicated that RBPs were conserved among different trypanosomes.

**Figure 5:**
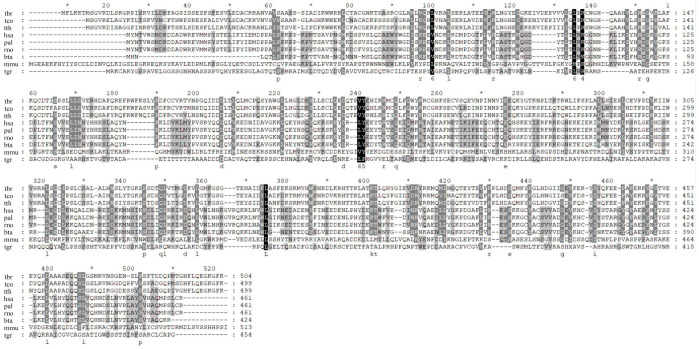
Multiple sequence alignment of Pi3K proteins among different species. The asterisk indicated the middle of two sites. The shadow indicated the similarity level in the site. Abbreviation for the species: bta, *Bos taurus*; hsa, *Homo sapiens*; mmu, *Mus musculus*; pal, *Pteropus alecto*; rno, *Rattus norvegicus*; tbr, *Trypanosoma brucei*; tco, *Trypanosoma conorhini*; tgr, *Trypanosoma grayi;* tth, *Trypanosoma theileri*.

Phosphatidylinositol-3 kinase (PI3K) is one of the most frequently activated pathogenic signaling proteins in human disease [41]. Two PI3Ks were identified in *T. brucei* by the sequence alignment method. Multiple sequence alignment of PI3K in various species conducted using Clustal Omega [42] suggested a very low similarity of PI3K between trypanosomes and mammals, while high similarities were observed among trypanosomes (Figure 5). These results suggest that PI3K might serve as a reliable drug target to control trypanosome.

### 3.5. miRNA Identification

The miRNAs are a type of conserved non-coding RNAs involved in post-translational regulation. Although many practical difficulties need to be overcome, the miRNA-mediated method showed a possible future treatment for some diseases [43]. Currently, no trypanosome miRNAs have been reported in the miRBase database; thus, a systemic identification of *T. brucei* miRNAs was needed. The EST-based method is an effective method for identifying novel miRNAs in published genomes [13,14]. By aligning the *T. brucei* genome to known miRNAs in the miRBase database. Attributes of these miRNAs, including length, EST accession and mismatch are listed in Table 3. The length of these miRNAs ranged from 18nt to 23nt. The mismatch value is an important parameter to discriminate miRNAs from other non-coding RNAs. One miRNA (tbr-miR-466i-5p) showed mismatch = 0, suggesting that this miRNA is identical to known miRNAs (mmu-miR-466i-5p). A set of 15 identified miRNAs showed only one base difference (mismatch = 1) with known miRNAs.

The identified 26 miRNAs were categorized into 23 different families based on the miRNA classification. Only three miRNA families (miR-3613, miR-466, miR-551) had more than one member (tbr-miR-3613 and tbr-miR-3613-5p; tbr-miR-466f-3p and tbr-miR-466i-5p; tbr-miR-551-3p and tbr-miR-551b-3p).

### 3.6. Homolog Distribution of Identified miRNAs

*T. brucei* can cause African trypanosomiasis, which is one of the neglected tropical diseases worldwide [44]. After searching in the miRBase database, 57.7% (15/26) of the *T. brucei* miRNAs were found to have homologous miRNAs in homo sapiens (human) genome (Table 4). Although *T. brucei* is a type of protozoan, it had fewer homologous miRNAs with Platyhelminthes (such as Schmidtea mediterranea) than mammals (such as human). Moreover, some miRNAs showed only one homology in other species, meaning that this type of miRNA was rare in animals. A set of 21 miRNAs had homology detected in mammals, while five miRNAs (tbr-miR-2491-3p, tbr-miR-752-3p, tbr-miR-8406-3p, tbr-miR-1599 and tbr-miR-4171-5p) were unique in *T. brucei*, with no homologs found in mammalian hosts. Interestingly, only one *T. brucei* miRNA (miR-2491-3p) was identified in tsetse fly but could not be found in human genome. Since tsetse fly is the vector of trypanosome and could transmit *T. brucei* to human, we hypothesized that this miRNA might be a critical molecule involved in the infection of *T. brucei*.

### 3.7. Network Analysis

**Figure 6:**
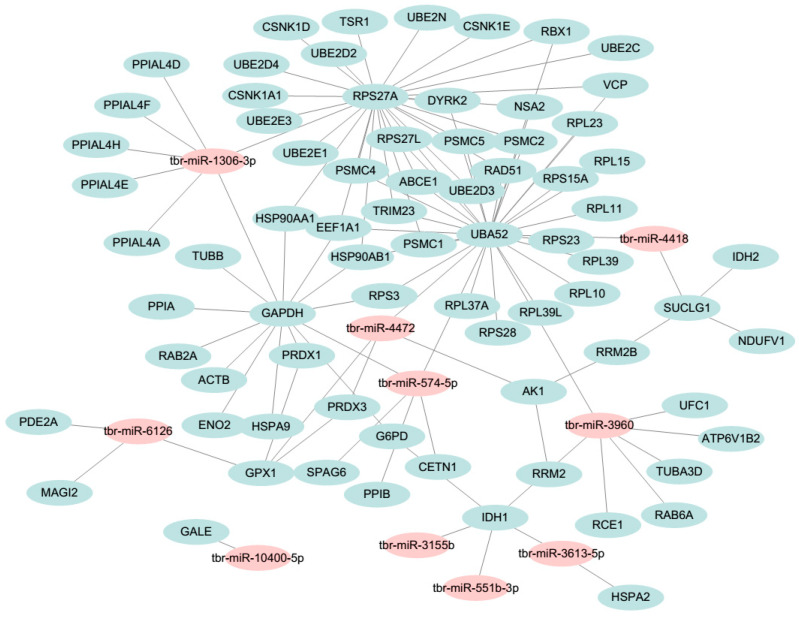
The miRNA–gene interaction sub-network. The pink node represents a miRNA, while the light blue node represents a gene. The full view of this network is shown in the Supplementary Materials.

To explore the regulatory mechanisms of miRNA in *T. brucei*, we constructed the miRNA-gene network using Cytoscape. The resulting network was composed of 15 miRNAs and 551 edges (Figure S2). Most genes were found to co-regulate by over two miRNAs and these genes were supposed to work systematically through co-regulated miRNAs in special biological processes. We then applied toolkit “NetworkAnalyzer” inside Cytoscape to calculate the betweenness centrality of each edge in the network (Table S1). We further selected the top edges to construct a sub-network (Figure 6). In the resulting sub-network, several key nodes were found: RPS27A, UBA52 and GAPDH. The RPS27A was reported to regulate proliferation, promote cell progression and inhibit apoptosis of leukemia cells. This gene might act as a controller of microglia activation in triggering neurodegeneration in trypanosome infection [45]. The UBA52 regulates ubiquitination of ribosome and sustains embryonic development, and is essential for the replication of virus–host interaction in chicken [46]. GAPDH is a multifunctional protein present both in eukaryotes and prokaryotes. This molecule has been reported to be correlated with redox status in trypanosome [47]. These results suggest that the identified nodes are critical molecules involved in the infection of *T. brucei*.

### 3.8. Drug Target Search

By searching potential drugs in Drugbank database [48], we speculated six candidates for treating *T. brucei* (Table 5). Some of these drugs are in the experiment period, while others have been approved by FDA for treatment of other diseases, such as Decitabine and Mebendazole. In this study, Decitabine was supposed to target Adenylate kinase (XP_827176.1) in *T. brucei*. Decitabine is a pyrimidine nucleoside analog used for the treatment of myelodysplastic syndromes by inducing alterations in gene expression [49]. Mebendazole was supposed to target Alpha tubulin (XP_001218940.1) by drug target analysis. Mebendazole is a benzimidazole anthelmintic used to treat parasite infections. Mebendazole acts by interfering with carbohydrate metabolism and inhibiting polymerization of microtubules [50]. We believe these drugs could also be used in the treatment of *T. brucei* infection.

## 4. Discussion

Tsetse flies are a type of blood-sucking insect that live in diverse locations in sub-Saharan Africa. They are insect vectors transmitting the parasite *T. brucei*. This parasite can cause a tropical disease called African trypanosomiasis. The treatment for this disease is not satisfying, exploring more detail about *T. brucei* genome is urgent [2]. With the development of bioinformatics technology, more and more tools have been developed, which offer an unprecedented opportunity for genome-wide identification of proteins and miRNAs in this parasite.

A set of 8758 proteins is reported in the current version ASM244v1 of *T. brucei* genome, among which 5709 protein functions remain unknown. The proteins of *T. brucei* and other trypanosomes were compared by sequence alignment method. The protein homolog proportion between two studied trypanosomes is larger than 80%, while the top proportion was found between *T. brucei* and *T. equiperdum*. However, when compared with the trypanosome and mammal genomes, the corresponding homolog proportion was extremely low. All the mammals shared less than 5% protein homology with *T. brucei*, meaning that the trypanosome is highly different from mammal in the genome aspect.

To investigate the possible evolution mechanism in the molecular function of this parasite, the protein homologs shared in *T. brucei* and mammals were studied. A set of 142 proteins were found to be present in *T. brucei* and mammalian genomes and these proteins were then denoted as TbrMam proteins. By Metascape enrichment analysis, we found that these TbrMam proteins were significantly enriched in related functions, such as “Cellular responses to stress” and “cell redox homeostasis”. This result suggested that *T. brucei* may use some proteins to acquire and transport essential nutrients from mammalian hosts. Trypanosome lives in the blood of mammal where high oxidative stress is present [51]. Thus, trypanosomes have evolved a strong antioxidant defense system to cope with these stressors. A previous study reported that trypanosomes have evolved a rapid and precise response toward oxidative stress and maintain redox homeostasis [52].

By ShinyGO genomic analysis, we have further found that these TbrMam genes were significantly enriched in four genomic aspects (number of exons, coding sequence length, GC content and 3′-UTR length). In addition, by comparing with proteins in other trypanosomes, we have also identified 32 novel pathogenic proteins (24 RBPs, 6 VSGs and 2 PI3K proteins) in *T. brucei*. These proteins were shared among different trypanosomes. These pathogenic proteins were supposed to be deeply involved in trypanosome–host interaction and could be used as drug targets in future studies.

Using the EST-based approach, we identified 26 new miRNAs in *T. brucei*. A set of 15 identified miRNAs showed only one base difference with known miRNAs, indicating that the results of our miRNA identification are highly reliable. By comparison with miRNAs in other animals, we found that human miRNAs shared the greatest number of *T. brucei* miRNAs, meaning that trypanosome share the similar miRNA evolution mechanism with human. Five miRNAs (tbr-miR-2491-3p, tbr-miR-752-3p, tbr-miR-8406-3p, tbr-miR-1599 and tbr-miR-4171-5p) were found to have no homologs with mammalian hosts. The miR-1599 was previously found specifically in chicken genome. The miR-1599 could bind to Toll-like receptors, which recognize diverse pathogen-associated molecular patterns and play a critical role in the immune response [53].

We then constructed a miRNA-gene network by Cytoscape. Network topological analysis suggested that several nodes (RPS27A, UBA52 and GAPDH) are located in the center of the network. These central nodes might serve as drug targets for trypanosome infection. Previous studies suggested that trypanosome depended entirely on glycolysis for the acquisition of energy supply [4]. Therefore, targeting its special energy metabolism is one of effective strategies to control trypanosome [54]. Among glycolysis, the GAPDH is the most important enzyme in glycolysis reaction. The GAPDH was suggested as a potential drug target and was validated in the previous study [55]. Inhibitors of trypanosome GAPDH may represent promising lead structures for the design of innovative anti-trypanosome agents.

## 5. Conclusions

We applied a series of bioinformatics tools to study the protein-coding genes and miRNAs in *T. brucei*. Through comparative analyses of *T. brucei* and mammalian genomes, we identified several novel proteins and miRNAs, which could be used as novel biomarkers for studying parasite–host interactions. These molecules could be applied as effective targets for future drug development of African trypanosomiasis.

## Figures and Tables

**Table 1:** Functional enrichment of TbrMam proteins by tool Metascape.

Enrichment Term	Category	Description	Count	Percent (%)	*p*-Value
R-HSA-2262752	Reactome Gene Sets	Cellular responses to stress	37	26.06	1.29 × 10^−26^
WP534	WikiPathways	Glycolysis and gluconeogenesis	9	6.34	6.61 × 10^−13^
R-HSA-1852241	Reactome Gene Sets	Organelle biogenesis and maintenance	16	11.27	8.71 × 10^−13^
R-HSA-6798695	Reactome Gene Sets	Neutrophil degranulation	18	12.68	1.48 × 10^−11^
GO:0006886	GO Biological Processes	intracellular protein transport	20	14.08	5.62 × 10^−11^
R-HSA-2132295	Reactome Gene Sets	MHC class II antigen presentation	10	7.04	3.63 × 10^−10^
GO:0010035	GO Biological Processes	response to inorganic substance	17	11.97	4.57 × 10^−10^
GO:0000278	GO Biological Processes	mitotic cell cycle	18	12.68	5.89 × 10^−10^
GO:0006091	GO Biological Processes	generation of precursor metabolites	15	10.56	6.31 × 10^−10^
GO:0045454	GO Biological Processes	cell redox homeostasis	6	4.23	3.24 × 10^−8^
GO:0006107	GO Biological Processes	oxaloacetate metabolic process	4	2.82	5.75 × 10^−8^
WP3888	WikiPathways	VEGFA-VEGFR2 signaling pathway	13	9.15	1.74 × 10^−7^

**Table 2:** The identified new pathogenic proteins in *T. brucei*. A total of 32 proteins were identified in this table.

*T. brucei* Protein	Identity (%)	BLAST e-Value	Protein Classification	Current Annotation
XP_844715.1	99.75	0	Invariant surface glycoprotein	hypothetical protein Tb927.5.410
XP_844716.1	99.73	0	Invariant surface glycoprotein	hypothetical protein Tb927.5.420
XP_951504.1	99.68	0	Invariant surface glycoprotein	hypothetical protein Tb927.2.1600
XP_823168.1	99.6	0	Invariant surface glycoprotein	hypothetical protein Tb10.6k15.0940
XP_844736.1	99.52	0	Invariant surface glycoprotein	hypothetical protein Tb927.5.620
XP_844705.1	82.44	0	Invariant surface glycoprotein	hypothetical protein Tb927.5.310
XP_846014.1	72.34	0	Phosphatidylinositol 3-kinase	hypothetical protein, conserved
XP_844230.1	61.46	0	Phosphatidylinositol 3-kinase	hypothetical protein, conserved
XP_844782.1	100	2.0 × 10^−174^	RNA-binding protein	hypothetical protein, conserved
XP_844136.1	100	0	RNA-binding protein	hypothetical protein, conserved
XP_845741.1	100	3.0 × 10^−113^	RNA-binding protein	hypothetical protein, conserved
XP_828127.1	100	0	RNA-binding protein	hypothetical protein, conserved
XP_823405.1	100	3.0 × 10^−163^	RNA-binding protein	hypothetical protein, conserved
XP_843699.1	100	0	RNA-binding protein	hypothetical protein, conserved
XP_844884.1	100	0	RNA-binding protein	hypothetical protein, conserved
XP_845763.1	100	4.0 × 10^−130^	RNA-binding protein	hypothetical protein, conserved
XP_827049.1	100	3.0 × 10^−100^	RNA-binding protein	uncharacterized protein Tb09.160.5020
XP_827838.1	100	0	RNA-binding protein	uncharacterized protein Tb10.05.0260
XP_829716.1	100	0	RNA-binding protein	uncharacterized protein Tb11.01.8310
XP_828145.1	100	1.0 × 10^−145^	RNA-binding protein	uncharacterized protein Tb11.03.0550
XP_827481.1	99.64	0	RNA-binding protein	hypothetical protein, conserved
XP_843794.1	99.54	2.0 × 10^−161^	RNA-binding protein	hypothetical protein, conserved
XP_823414.1	81.98	2.0 × 10^−66^	RNA-binding protein	uncharacterized protein Tb10.389.1650
XP_829659.1	73.57	0	RNA-binding protein	uncharacterized protein Tb11.01.7680
XP_823415.1	71.19	2.0 × 10^−55^	RNA-binding protein	uncharacterized protein Tb10.389.1640
XP_844504.1	67.58	2.0 × 10^−161^	RNA-binding protein	hypothetical protein, conserved
XP_827841.1	100	0	RNA-binding protein 27	uncharacterized protein Tb10.05.0020
XP_827855.1	100	0	RNA-binding protein 29	uncharacterized protein Tb10.61.3200
XP_828416.1	100	0	RNA-binding protein 34	hypothetical protein, conserved
XP_828660.1	99.79	0	RNA-binding protein 38	uncharacterized protein Tb11.02.3610
XP_845561.1	100	0	RNA-binding protein 42	hypothetical protein, conserved
XP_829150.1	63.05	0	RNA-binding protein NOB1	hypothetical protein, conserved

**Table 3:** Identified novel miRNAs in *T. brucei*. Most of the sequences are similar to known miRNAs.

No.	TBR miRNA	miRNA Sequence	Length	EST Accession	Mismatch
1	tbr-miR-466i-5p	TGTGTGTGTGTGTGTGTGTG	20	W04105.1	0
2	tbr-miR-1277-5p	TATATATATATATGTACGTAT	21	AI215308.1	1
3	tbr-miR-1599	GGAGGGAGGAAAAAAAAAAA	20	W06624.1	1
4	tbr-miR-2444	TTTGTGTTGTTTTTTGTTTT	20	AI510924.1	1
5	tbr-miR-2491-3p	CAACAACAGCAGCAGCAA	18	AA114338.1	1
6	tbr-miR-3613	TGTTGTACTTTTTTTTTTGT	20	AI510925.1	1
7	tbr-miR-3960	GGCGGCGGCGGAGGCGGGGG	20	BE040956.1	1
8	tbr-miR-4171-5p	TGACTCTCTTAAGGAAGCCA	20	AJ234107.1	1
9	tbr-miR-466f-3p	CATACACACACACATACACAC	21	AA689173.1	1
10	tbr-miR-467g	TATACATACACACACATATAT	21	AI707381.1	1
11	tbr-miR-551-3p	GCGACCCATACTTGGTTTCA	20	BU792216.1	1
12	tbr-miR-551b-3p	GCGACCCATACTTGGTTTCA	20	BU792216.1	1
13	tbr-miR-626	AGCTGTCTGAAAATGTCTT	19	AA736241.1	1
14	tbr-miR-752-3p	AGTCAGCATTGGTGGTTT	18	AA843025.1	1
15	tbr-miR-8406-3p	TTTTCTTAAGATTTATTTTG	20	W40052.1	1
16	tbr-miR-8485	CACACACACACACACACGTAT	21	W04025.1	1
17	tbr-miR-10400-5p	CGGCGGCGGCGGCTCTGGGCG	21	CO724197.1	2
18	tbr-miR-1306-3p	ACGTTGGCTCTGGTGGTG	18	DR752331.1	2
19	tbr-miR-3155b	CCAGGCTCTGCAGTGGGA	18	AI374163.1	2
20	tbr-miR-3613-5p	TGTTGTACTTTTTTTTTTGTTC	22	AI510925.1	2
21	tbr-miR-4308	TCCCTGGAGTTTCTTCTT	18	W84094.1	2
22	tbr-miR-4418	CACTGCAGGACTCAGCAG	18	T26203.1	2
23	tbr-miR-4472	GGTGGGGGGTGTTGTTTT	18	AI215262.1	2
24	tbr-miR-4711-3p	CGTGTCTTCTGGCTTGAT	18	AA003459.1	2
25	tbr-miR-574-5p	TGAGTGTGTGTGTGTGAGTGTGT	23	N99275.1	2
26	tbr-miR-6126	GTGAAGGCCCGGCGGAGA	18	W06685.1	2

**Table 4:** Homolog distribution of identified miRNA when compared to other species. The block “YES” indicated that the homolog of corresponding miRNA was present in the corresponding species. The blue block indicated the mammals, while yellow block indicated the non-mammal species. Abbreviation of the species: bta, *Bos Taurus*; cli, *Columba livia*; cin, *Ciona intestinalis*; cpo, *Cavia porcellus*; dme, *Drosophila melanogaster*; gga, *Gallus gallus*; hsa, *Homo sapiens*; mmu, *Mus musculus*; sma, *Schistosoma mansoni*; sme, *Schmidtea mediterranea*; ssc, *Sus scrofa*; tch, *Tupaia chinensis*.

TBR miRNA	Mammal					Non-Mammal						
	hsa	mmu	bta	cpo	ssc	gga	sma	sme	tch	dme	cli	cin
miR-10400-5p	YES	-	-	-	-	-	-	-	-	-	-	-
miR-1277-5p	YES	-	-	YES	-	-	-	-	YES	-	-	-
miR-1306-3p	YES	YES	-	-	YES	YES	-	-	YES	-	YES	-
miR-1599	-	-	-	-	-	YES	-	-	-	-	-	-
miR-2444	-	-	YES	-	-	-	-	-	-	-	-	-
miR-2491-3p	-	-	-	-	-	-	-	-	-	YES	-	-
miR-3155b	YES	-	-	-	-	-	-	-	-	-	-	-
miR-3613	-	-	-	-	YES	-	-	-	-	-	-	-
miR-3613-5p	YES	-	-	YES	-	-	-	-	-	-	-	-
miR-3960	YES	YES	-	-	-	-	-	-	-	-	-	-
miR-4171-5p	-	-	-	-	-	-	-	-	-	-	-	YES
miR-4308	YES	-	-	-	-	-	-	-	-	-	-	-
miR-4418	YES	-	-	-	-	-	-	-	-	-	-	-
miR-4472	YES	-	-	-	-	-	-	-	-	-	-	-
miR-466f-3p	-	YES	-	-	-	-	-	-	-	-	-	-
miR-466i-5p	-	YES	-	-	-	-	-	-	-	-	-	-
miR-467g	-	YES	-	-	-	-	-	-	-	-	-	-
miR-4711-3p	YES	-	-	-	-	-	-	-	-	-	-	-
miR-551-3p	-	-	-	YES	-	YES	-	-	-	-	-	-
miR-551b-3p	YES	YES	-	-	-	-	-	-	-	-	YES	-
miR-574-5p	YES	YES	-	YES	YES	-	-	-	-	-	-	-
miR-6126	YES	-	-	-	-	-	-	-	-	-	-	-
miR-626	YES	-	-	-	-	-	-	-	-	-	-	-
miR-752-3p	-	-	-	-	-	-	-	YES	-	-	-	-
miR-8406-3p	-	-	-	-	-	-	YES	-	-	-	-	-
miR-8485	YES	-	-	-	-	-	-	-	-	-	-	-

**Table 5:** Drug target analysis of *T. brucei* proteins.

No.	*T. Brucei* Accession	*T. Brucei* Protein	Predicted Drug	Accession	Chemical Formula
1	XP_827176.1	Adenylate kinase	Decitabine	DB01262	C8H12N4O4
2	XP_822929.1	Glyceraldehyde 3-phosphate dehydrogenase	D-glyceraldehyde 3-phosphate	DB02263	C3H7O6P
3	XP_951525.1	Small GTP-binding protein RAB6	Guanosine-5′-Diphosphate	DB04315	C10H15N5O11P2
4	XP_846100.1	Cyclophilin-type peptidyl-prolyl isomerase	1,4-Dithiothreitol	DB04447	C4H10O2S2
5	XP_829056.1	Polyubiquitin	N-Formylmethionine	DB04464	C6H11NO3S
6	XP_001218940.1	Alpha tubulin	Mebendazole	DB00643	C16H13N3O3

## Data Availability

All data are available from the NCBI genome database (gene), UniProt database (protein) and miRBase database (miRNA).

## Supplementary Materials

The following supporting information can be downloaded at: https://www.mdpi.com/article/10.3390/insects13110968/s1, Figure S1: The percentage of protein similarity between *T. brucei* and human; Figure S2: Full view of miRNA–gene interaction network; Table S1: TbrMam protein list; Table S2: betweenness centrality of edge in miRNA–gene interaction network.

## References

[1] Rogerson E., Pelletier J., Acosta-Serrano A., Rose C., Taylor S., Guimond S., Lima M., Skidmore M., Yates E. (2018). Variations in the Peritrophic Matrix Composition of Heparan Sulphate from the Tsetse Fly, Glossina morsitans morsitans. Pathogens.

[2] World Health Organization (2022). Trypanosomiasis, Human African (Sleeping Sickness). https://www.who.int/news-room/fact-sheets/detail/trypanosomiasis-human-african-.

[3] Naguleswaran A., Doiron N., Roditi I. (2018). RNA-Seq analysis validates the use of culture-derived Trypanosoma brucei and provides new markers for mammalian and insect life-cycle stages. BMC Genom..

[4] Smith T.K., Bringaud F., Nolan D.P., Figueiredo L.M. (2017). Metabolic reprogramming during the Trypanosoma brucei life cycle. F1000Research.

[5] Fall F., Mamede L., Schioppa L., Ledoux A., De Tullio P., Michels P., Frédérich M., Quetin-Leclercq J. (2022). Trypanosoma brucei: Metabolomics for analysis of cellular metabolism and drug discovery. Metabolomics.

[6] Jackson A.P., Sanders M., Berry A., McQuillan J., Aslett M.A., Quail M.A., Chukualim B., Capewell P., MacLeod A., Melville S.E. (2010). The Genome Sequence of *Trypanosoma brucei* gambiense, Causative Agent of Chronic Human African Trypanosomiasis. PLoS Negl. Trop. Dis..

[7] Parsons M., Ramasamy G., Vasconcelos E.J., Jensen B.C., Myler P.J. (2015). Advancing *Trypanosoma brucei* genome annotation through ribosome profiling and spliced leader mapping. Mol. Biochem. Parasitol..

[8] Glockzin K., Meek T.D., Katzfuss A. (2022). Characterization of adenine phosphoribosyltransferase (APRT) activity in *Trypanosoma brucei brucei*: Only one of the two isoforms is kinetically active. PLoS Negl. Trop. Dis..

[9] Kanehisa M., Sato Y. (2020). KEGG Mapper for inferring cellular functions from protein sequences. Protein Sci..

[10] Yang Z., Wu Y. (2019). Improved annotation of *Lutzomyia longipalpis* genome using bioinformatics analysis. PeerJ.

[11] Pkna B., Jd A., Srm A., Mg C., Nma C., Msa C., Vk D., Vr A. (2021). Functional annotation and sequence-structure characterization of a hypothetical protein putatively involved in carotenoid biosynthesis in microalgae. South Afr. J. Bot..

[12] Acuna S.M., Floeter-Winter L.M., Muxel S.M. (2020). MicroRNAs: Biological Regulators in Pathogen-Host Interactions. Cells.

[13] Yang Z., Wang M., Zeng X., Wan A.T.-Y., Tsui S.K.-W. (2020). In silico analysis of proteins and microRNAs related to human African trypanosomiasis in tsetse fly. Comput. Biol. Chem..

[14] Ahsan M.I., Chowdhury M.S.R., Das M., Akter S., Roy S., Sharma B., Akhand R.N., Hasan M., Uddin M.B., Ahmed S.S.U. (2021). In silico identification and functional characterization of conserved miRNAs in the genome of *Cryptosporidium parvum*. Bioinform. Biol. Insights.

[15] UniProt C. (2021). UniProt: The universal protein knowledgebase in 2021. Nucleic Acids Res..

[16] Kozomara A., Birgaoanu M., Griffiths-Jones S. (2019). miRBase: From microRNA sequences to function. Nucleic Acids Res..

[17] Yang Z., Tsui K.-W.S. (2018). Functional Annotation of Proteins Encoded by the Minimal Bacterial Genome Based on Secondary Structure Element Alignment. J. Proteome Res..

[18] Zhou Y., Zhou B., Pache L., Chang M., Khodabakhshi A.H., Tanaseichuk O., Benner C., Chanda S.K. (2019). Metascape provides a biologist-oriented resource for the analysis of systems-level datasets. Nat. Commun..

[19] Ge S.X., Jung D.M., Yao R.A. (2020). ShinyGO: A graphical gene-set enrichment tool for animals and plants. Bioinformatics.

[20] Zhang N., Jiang N., Zhang K., Zheng L., Zhang D., Sang X., Feng Y., Chen R., Yang N., Wang X. (2020). Landscapes of Protein Posttranslational Modifications of African Trypanosoma Parasites. iScience.

[21] Erben E., Leiss K., Liu B., Gil D.I., Helbig C., Clayton C. (2021). Insights into the functions and RNA binding of Trypanosoma brucei ZC3H22, RBP9 and DRBD7. Parasitology.

[22] Pereira S.S., Jackson A.P., Figueiredo L.M. (2021). Evolution of the variant surface glycoprotein family in African trypanosomes. Trends Parasitol..

[23] Schoijet A.C., Sternlieb T., Alonso G.D. (2017). The Phosphatidylinositol 3-kinase Class III Complex Containing TcVps15 and TcVps34 Participates in Autophagy in *Trypanosoma cruzi*. J. Eukaryot. Microbiol..

[24] Zhu Y., Zhu L., Wang X., Jin H. (2022). RNA-based therapeutics: An overview and prospectus. Cell Death Dis..

[25] Diener C., Keller A., Meese E. (2022). Emerging concepts of miRNA therapeutics: From cells to clinic. Trends Genet..

[26] Langmead B., Wilks C., Antonescu V., Charles R. (2019). Scaling read aligners to hundreds of threads on general-purpose processors. Bioinformatics.

[27] Gruber A.R., Bernhart S.H., Lorenz R. (2015). The ViennaRNA Web Services. RNA Bioinform..

[28] Sticht C., De La Torre C., Parveen A., Gretz N. (2018). miRWalk: An online resource for prediction of microRNA binding sites. PLoS ONE.

[29] Huang H.Y., Lin Y.C.D., Cui S.D., Huang Y.X., Tang Y., Xu J.T., Bao J.Y., Li Y.L., Wen J., Zuo H.L. (2022). miRTarBase update 2022: An informative resource for experimentally validated miRNA-target interactions. Nucleic Acids Res..

[30] Franz M., Lopes C.T., Huck G., Dong Y., Sumer O., Bader G.D. (2016). Cytoscape. js: A graph theory library for visualisation and analysis. Bioinformatics.

[31] World Health Organization (2022). Sleeping Sickness Elimination Progresses in 2021 Despite COVID-19. https://www.who.int/news/item/30-05-2022-sleeping-sickness-elimination-progresses-in-2021-despite-covid-19.

[32] Butenko A., Kostygov A.Y., Sadlova J., Kleschenko Y., Becvar T., Podesvova L., Macedo D.H., Zihala D., Lukes J., Bates P.A. (2019). Comparative genomics of Leishmania (Mundinia). BMC Genom..

[33] Yang Z., Hu F. (2018). Investigation of gene evolution in vertebrate genome reveals novel insights into spine study. Gene.

[34] Caljon G., Mabille D., Stijlemans B., De Trez C., Mazzone M., Tacchini-Cottier F., Malissen M., Van Ginderachter J.A., Magez S., De Baetselier P. (2018). Neutrophils enhance early Trypanosoma brucei infection onset. Sci. Rep..

[35] Stauffert D., Silveira M.F.d., Mesenburg M.A., Manta A.B., Dutra A.d.S., Bicca G.L.d.O., Villela M.M. (2017). Prevalence of *Trypanosoma cruzi*/HIV coinfection in southern Brazil. Brazilian J. Infect. Dis..

[36] Almeida E.A.d., Ramos Júnior A.N., Correia D., Shikanai-Yasuda M.A. (2011). Co-infection *Trypanosoma cruzi*/HIV: Systematic review (1980–2010). Rev. Soc. Bras. Med. Trop..

[37] Dolcini G.L., Solana M.E., Andreani G., Celentano A.M., Parodi L.M., Donato A.M., Elissondo N., Gonzalez Cappa S.M., Giavedoni L.D. (2008). *Trypanosoma cruzi* (Chagas’ disease agent) reduces HIV-1 replication in human placenta. Retrovirology.

[38] Sangenito L.S., Menna-Barreto R.F., Oliveira A.C., d’Avila-Levy C.M., Branquinha M.H., Santos A.L. (2018). Primary evidence of the mechanisms of action of HIV aspartyl peptidase inhibitors on *Trypanosoma cruzi* trypomastigote forms. Int. J. Antimicrob. Agents.

[39] Garrison P., Khan U., Cipriano M., Bush P.J., McDonald J., Sur A., Myler P.J., Smith T.K., Hajduk S.L., Bangs J.D. (2021). Turnover of Variant Surface Glycoprotein in *Trypanosoma brucei* Is a Bimodal Process. mBio.

[40] Romagnoli B.A., Holetz F.B., Alves L.R., Goldenberg S. (2020). RNA binding proteins and gene expression regulation in *Trypanosoma cruzi*. Front. Cell. Infect. Microbiol..

[41] Elmenier F.M., Lasheen D.S., Abouzid K.A.M. (2019). Phosphatidylinositol 3 kinase (PI3K) inhibitors as new weapon to combat cancer. Eur. J. Med. Chem..

[42] Sievers F., Higgins D.G. (2018). Clustal Omega for making accurate alignments of many protein sequences. Protein Sci..

[43] Saliminejad K., Khorshid H.R.K., Fard S.S., Ghaffari S.H. (2019). An overview of microRNAs: Biology, functions, therapeutics, and analysis methods. J. Cell. Physiol..

[44] World Health Organization (2022). Neglected Tropical Diseases. https://www.who.int/health-topics/neglected-tropical-diseases.

[45] Khayer N., Mirzaie M., Marashi S.-A., Jalessi M. (2020). Rps27a might act as a controller of microglia activation in triggering neurodegenerative diseases. PLoS ONE.

[46] Wang Q., Li Q., Liu T., Chang G., Sun Z., Gao Z., Wang F., Zhou H., Liu R., Zheng M. (2018). Host interaction analysis of PA-N155 and PA-N182 in chicken cells reveals an essential role of UBA52 for replication of H5N1 avian influenza virus. Front. Microbiol..

[47] Pariona-Llanos R., Pavani R.S., Reis M., Noel V., Silber A.M., Armelin H.A., Cano M.I.N., Elias M.C. (2015). Glyceraldehyde 3-phosphate dehydrogenase-telomere association correlates with redox status in *Trypanosoma cruzi*. PLoS ONE.

[48] Wishart D.S., Feunang Y.D., Guo A.C., Lo E.J., Marcu A., Grant J.R., Sajed T., Johnson D., Li C., Sayeeda Z. (2018). DrugBank 5.0: A major update to the DrugBank database for 2018. Nucleic Acids Res..

[49] Thota S., Oganesian A., Azab M., Griffiths E.A. (2021). Role of cedazuridine/decitabine in the management of myelodysplastic syndrome and chronic myelomonocytic leukemia. Future Oncol..

[50] Chai J.-Y., Jung B.-K., Hong S.-J. (2021). Albendazole and mebendazole as anti-parasitic and anti-cancer agents: An update. Korean J. Parasitol..

[51] Fullerton M., Singha U.K., Duncan M., Chaudhuri M. (2015). Down regulation of Tim50 in Trypanosoma brucei increases tolerance to oxidative stress. Mol. Biochem. Parasitol..

[52] Mesías A.C., Garg N.J., Zago M.P. (2019). Redox balance keepers and possible cell functions managed by redox homeostasis in *Trypanosoma cruzi*. Front. Cell. Infect. Microbiol..

[53] Gholizade M., Fayazi J., Rahimnahal S. (2019). Prediction of MicroRNAs bind to Toll-like Receptors Pathway in Chicken based on Bioinformatics Method. Res. Mol. Med..

[54] Herrmann F.C., Lenz M., Jose J., Kaiser M., Brun R., Schmidt T.J. (2015). In silico identification and in vitro activity of novel natural inhibitors of Trypanosoma brucei glyceraldehyde-3-phosphate-dehydrogenase. Molecules.

[55] Cáceres A.J., Michels P.A., Hannaert V. (2010). Genetic validation of aldolase and glyceraldehyde-3-phosphate dehydrogenase as drug targets in Trypanosoma brucei. Mol. Biochem. Parasitol..

