# Supplementary figures and images for: Genome-Wide Screening for Pathogenic Proteins and microRNAs Associated with Parasite–Host Interactions in Trypanosoma brucei
Source: Insects. 2022 Oct 22;13(11):968. doi: 10.3390/insects13110968 (PMC9695099; doi:10.3390/insects13110968)

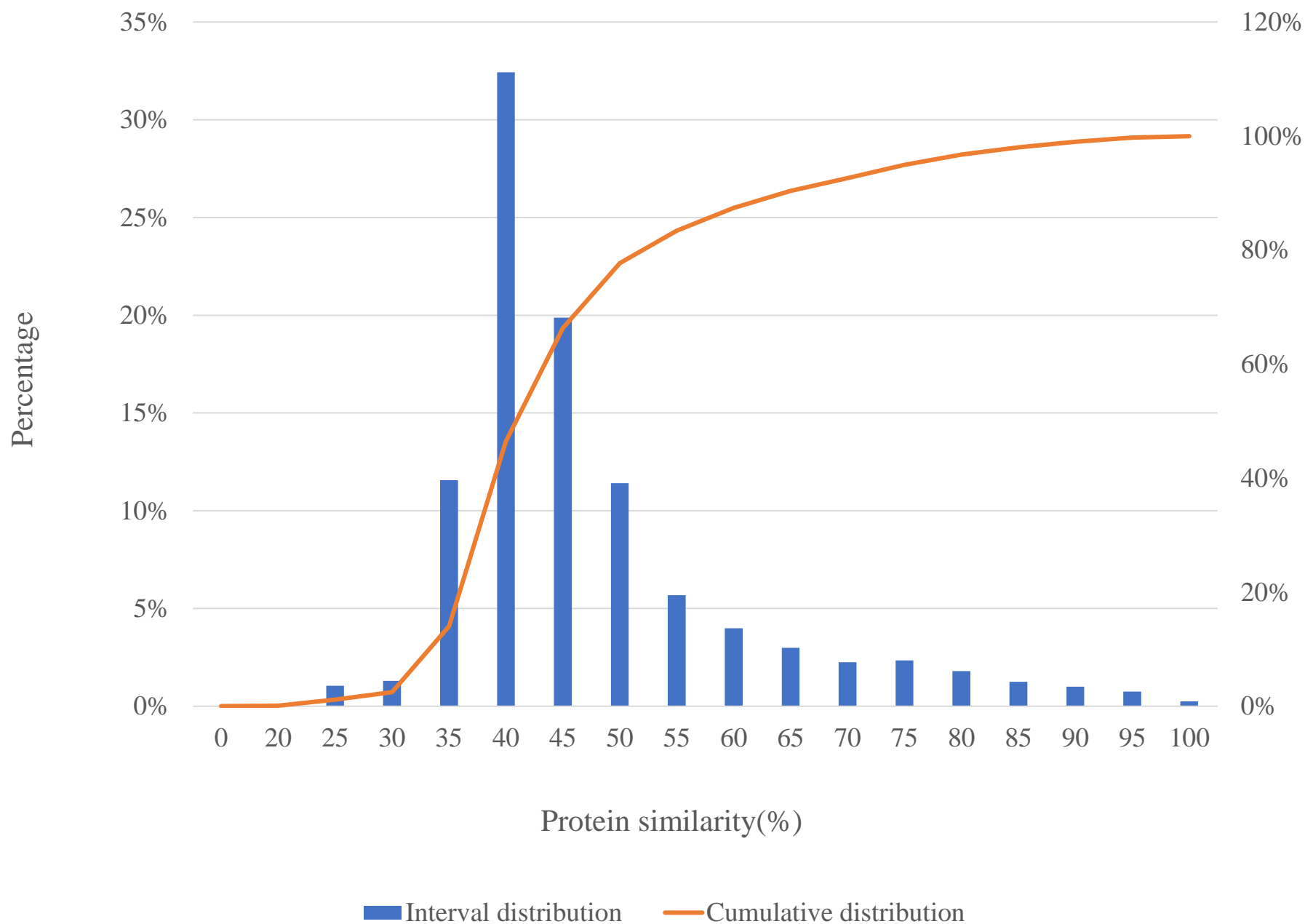

Supplement: Supplementary file 1 [file insects-13-00968-s001.zip › insects-1954268-supplementary-minor - upload/Figure S1 - The percentage of protein similarity between T. brucei and human.pdf]

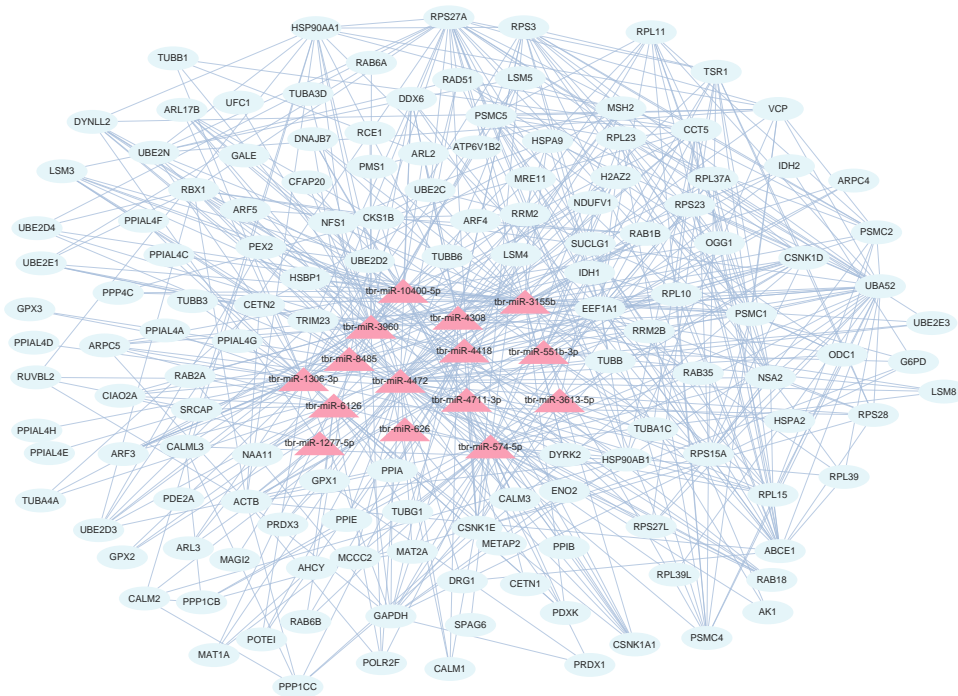

Supplement: Supplementary file 1 [file insects-13-00968-s001.zip › insects-1954268-supplementary-minor - upload/Figure S2 - Full view of miRNA gene network.pdf]
